# A Sparse Volume Reconstruction Method for Fetal Brain MRI Using Adaptive Kernel Regression

**DOI:** 10.1155/2021/6685943

**Published:** 2021-03-05

**Authors:** Qian Ni, Yi Zhang, Tiexiang Wen, Ling Li

**Affiliations:** ^1^Shenzhen Hospital of Guangzhou University of Chinese Medicine, Shenzhen, China; ^2^Radiology & Vascular Surgery, Department of Radiology, Zhongda Hospital, Medical School, Southeast University, Nanjing, China; ^3^Shenzhen Institutes of Advanced Technology, Chinese Academy of Sciences, Shenzhen, China; ^4^National Innovation Center for Advanced Medical Devices, Shenzhen, China; ^5^Suzhou Institute of Advanced Technology, Chinese Academy of Sciences, Suzhou, China

## Abstract

Slice-to-volume reconstruction (SVR) method can deal well with motion artifacts and provide high-quality 3D image data for fetal brain MRI. However, the problem of sparse sampling is not well addressed in the SVR method. In this paper, we mainly focus on the sparse volume reconstruction of fetal brain MRI from multiple stacks corrupted with motion artifacts. Based on the SVR framework, our approach includes the slice-to-volume 2D/3D registration, the point spread function- (PSF-) based volume update, and the adaptive kernel regression-based volume update. The adaptive kernel regression can deal well with the sparse sampling data and enhance the detailed preservation by capturing the local structure through covariance matrix. Experimental results performed on clinical data show that kernel regression results in statistical improvement of image quality for sparse sampling data with the parameter setting of the structure sensitivity 0.4, the steering kernel size of 7 × 7 × 7 and steering smoothing bandwidth of 0.5. The computational performance of the proposed GPU-based method can be over 90 times faster than that on CPU.

## 1. Introduction

Magnetic resonance imaging (MRI) is an ideal diagnostic technique for researchers to investigate the development of the fetal brain [1]. Its advantages are the absence of ionizing radiation, the availability of different contrast options (T1-weighted, T2-weighted, and diffusion-weighted imaging), and the superior contrast of soft tissue compared with ultrasonography, and MRI is also a safe and noninvasive procedure for patients and fetuses [2–4]. For these reasons, MRI has been widely used to investigate the developing fetal brain in vivo [5]. For fetal brain MRI, the high-quality volume representation of 3D acquisition has significant clinical meaning [6]. By the observation of the reconstructed volume data, researchers can study the mechanism of brain development and maturation [7] and identify the fetal brain abnormality or potential injury [8, 9], such as brain tumors, vascular malformations, and posterior fossa abnormalities. Fetal brain MRI can provide abundant information about aid clinical management, prognostication, and counseling [10].

The duration of an examination is typically 45 to 60 minutes for fetal brain MRI [1]. One major problem of fetal brain MRI is motion artifacts caused by fetal and maternal motion, because of the long acquisition times of 3D MRI scanning. Maternal motion may be avoided by some measures, but fetal motion is usually fast and unpredictable, especially for the younger fetus. Thus, it is still challenging to reconstruct high-fidelity image for fetal brain MRI due to the presence of fetal motion. For fetal motion, different strategies can be adopted to reduce the motion artifacts on MRI [11]. The first strategy tries to prevent the motion occurring during the examination, such as maternal sedation. The second one tries to quicken the data sampling speed. The faster the acquisition techniques for fetal brain MRI are, the lower the motion occurs. For example, the single-shot fast spin echo (SSFSE) T2-weighted imaging can acquire a slice at 1-second speed [12]. On the other hand, sparse data sampling technique can be applied to shorten the time of data acquisition. The last strategy tries to reconstruct high-quality image through advanced postprocessing motion detection and correction algorithms, such as the SVR method [13].

For the SVR framework [14], it includes the following steps to reduce the fetal motion and reconstruct the high-quality 3D result: motion identification and exclusion step, registration step, reconstruction step, and regularization step. For the motion identification and exclusion step, we should estimate the amount of motion and exclude the slices with large amount of motion corruption. Early reconstruction approaches need to manually exclude the motion corrupted slices. The intersection-based motion correction approach can automatically detect and reject motion corrupted and incorrect registration slices by the abnormal level of their mean squared intensity difference with respect to all other intersecting slices [15]. In [16], Kainz et al. have proposed an approach to automatically estimate the amount of motion based on the low-rank decomposition for linearly correlated image slices [17]. Using this approach, we can reject stacks with large motion and choose the stack with the least motion as the template to prepare for the registration step. Registration step can be utilized to correct the motion between slice and the reconstructed volume. Rousseau et al. [18] combined the 2D/3D registration with the PSF to achieve the 3D reconstruction. PSF [14] is a mathematical function to model the actual appearance of data points in physical space. By PSF, we can physically correct estimation of the image acquisition process. Subsequently, the SVR method was modified to improve the robustness of the 2D/3D registration [19]. For the reconstruction step, superresolution methods [20, 21] are utilized to reconstruct the 3D volume. In [22], Gholipour and Warfield combined the superresolution method with slice-to-volume registration to reduce the burring effect. Because the motion identification and exclusion steps can exclude the slice of which the motion amount is greater than the threshold, the amounts of the slightly corrupted slices are still preserved for reconstruction. Using the robust superresolution volume reconstruction method [23], the weight of slightly corrupted and misaligned slices would be reduced to minimize the effect of motion. During the process of superresolution reconstruction, maximum likelihood estimation (MLE) is treated as an optimum solution to estimate the point's value [24]. To get better results, we should minimize the difference between the estimated slices and the acquired slices. Since the minimization only depends on the acquired samples, the estimation in the MLE framework is ill-posed and inaccurate when the samples are sparse [23]. The regularization step is used to solve the overfitting problem, and it can reduce image noise and registration errors. In [25], Charbonnier et al. proposed a deterministic edge-preserving regularization method to deal with image. However, this method makes it difficult to avoid the smoothing of edges. Adaptive regularization techniques can be employed to reduce the smoothing effects of regularization [26]. In [27], Rousseau et al. took advantage of total variation regulation to extend the superresolution reconstruction method.

The general SVR framework with the superresolution reconstruction method has been developed in [28]. One important way to alleviate fetal motion is to quicken the data acquisition time by the sparse data sampling technique. However, the traditional SVR method could not deal well with the sparse sampling problem and cannot provide high-quality image. In this paper, we utilize the SVR method with adaptive kernel regression to cope with the sparse volume reconstruction with minimum motion artifacts under the condition of sparse data acquisition. The key improvements compared to previous works are as follows: firstly, we make use of the sparse samples to get faster speed of data acquisition in fetal brain MRI. Next, the adaptive kernel regression-based reconstruction method [29] with robust statistics calculation [24] can reconstruct high-quality volume under the condition of sparse sampling. In general, our comprehensive reconstruction method for fetal brain MRI mainly includes slice-to-volume registration, the robust statistics calculation, the PSF-based volume update, and adaptive kernel regression-based volume update.

The rest of the paper is organized as follows. The detailed methodology is discussed in Section 2. We design the actual implementation of the algorithm in Section 3.  Section 4 involves the experiment results and compares with those of superresolution methods. In this section, we also discuss how to determine the optimal values of related parameters using GPU-based fast reconstruction. Finally, we make a brief conclusion in Section 5.

## 2. Methods

### 2.1. Model of Data Acquisition and Motion Estimation

During data acquisition of fetal brain MRI, we collected several stacks of 2D slices in different orientations. Because of the fetal motion, the movement could be observed between these slices. Assume that the acquired *k* misaligned 2D slices are *I*_*j*_ ∈ **R**^*n*×*h*^, *j* = 1, ⋯, *k*, and the corresponding sparse 2D slices are *I*_*j*_^*s*^ ∈ **R**^*n*×*h*^, *j* = 1, ⋯, *k*. During the slice acquisitions of MRI, the inhomogeneity of the magnetic field *B*_*j*_, *j* = 1, ⋯, *k*, affects the intensities of the slices and the scaling factor *S*_*j*_′, *j* = 1, ⋯, *k*, is potentially different for each acquired slices. In [30], the logarithmic transformation was chosen to make the bias additive. However, field in-homogeneities are known to be multiplicative. Differently, we use the multiplicative bias field to form the multiplicative exponential model which replaces the logarithmic model. So the scaled and bias corrected slice *I*_*j*_′ can be modeled as

(1) $$\begin{array}{c} I_{j}^{s}=\text{sparse}\left(I_{j}\right),\\ \text{vec}\left(I_{j}^{\prime }\right)=S_{j}^{\prime }\cdot \exp\left(-B_{j}\right)\cdot \text{vec}\left(I_{j}^{s}\right), \end{array}$$

where *I*_*j*_^*s*^ is the sparsely sampled slice coming from the sparse operator sparse(∙), vec(∙) is the vectorization operator that transforms a *m*-pixel (*m* = *n* × *h*) image **R**^*n*×*h*^ into a vector of intensity values **R**^*m*^. The corresponding *k*-aligned 2D ground-truth slices are *I*_*j*_^∗^ ∈ **R**^*n*×*h*^, *j* = 1, ⋯, *k*. The relationship between corrected slices *I*_*j*_′ and the ground-truth slices *I*_*j*_^∗^ can be denoted as follows:

(2) $$\begin{array}{c} \text{vec}\left(I_{j}^{\prime }\right)=\theta_{j}\cdot \text{vec}\left(I_{j}^{*}\right)+\text{vec}\left(e_{j}\right),\;j=1,\cdots ,k, \end{array}$$

where *e*_*j*_ is the motion error, and *θ*_*j*_ denotes the unknown motion transformation parameter of slice *I*_*j*_^∗^. Then, we can define the following data matrix:

(3) $$\begin{array}{c} D=\left[\text{vec}\left(I_{1}^{\prime }\right);\cdots ;\text{vec}\left(I_{k}^{\prime }\right)\right]\in R^{m\times k},\\ X=\left[\text{vec}\left(I_{1}^{*}\right);\cdots ;\text{vec}\left(I_{k}^{*}\right)\right]\in R^{m\times k},\\ E=\left[\text{vec}\left(e_{1}\right);\cdots ;\text{vec}\left(e_{k}\right)\right]\in R^{m\times k},\\ T_{\text{total}}=\left[\theta_{1};\cdots ;\theta_{k}\right]\in R^{m\times k}. \end{array}$$

where *D*, *X*, *E*, and *T*_total_ denote the observed data matrix, reconstructed data matrix, motion error matrix, and the rigid transformation matrix. Given these definitions, the observed data matrix *D* can be described as *D* = *T*_total_∙*X* + *E*. The motion error matrix *E* is mainly caused by misaligned slices. The misaligned slices can cause the inaccurate reconstructed volume, and we want to exclude the stack which has many misaligned slices. However, we cannot directly calculate the amount of stack motions for the observed data matrix *D*, but a low-rank approximation *D*^∗^ as surrogate estimate can be used to evaluate the stack motion indirectly [16]. It has been shown that *D*^∗^ provides the best approximation to *D* [31]. The difference value between *D*^∗^ and *D* measures the motion error *E*. The smaller difference value indicates that the stack has fewer motions. To provide the low-rank approximation, the singular value decomposition is used to decompose the data matrix *D* as *D*_*m*×*k*_ = *U*_*m*×*k*_*S*_*k*×*k*_*V*_*k*×*k*_^*T*^. The singular value decomposition of *D* produces three matrices *U*, *S*, and *V*. *U* and *V* are both orthogonal matrices, and *S* is the diagonal matrix containing the singular values on the diagonal. And the singular value decomposition of *D*^∗^ is the first *r* singular values of the original matrix *D*, i.e., *D*_*m*×*k*_^∗^ = *U*_*m*×*r*_^∗^*S*_*r*×*r*_^∗^*V*_*r*×*k*_^∗*T*^, *r* = 1, ⋯, *k*. *U*^∗^ and *V*^∗^ are the first *r* columns of *U* and *V*, and *S*^∗^ is the top left *r* × *r* submatrix of *S*. The relative error based on the Frobenius norm ‖*D* − *D*^∗^‖ is used to measure the approximation between *D*^∗^ and *D*, i.e. *δ*_*r*_ = ‖*D* − *D*_*r*_^∗^‖/‖*D*‖. For the different values of *r* = 1, ⋯, *k*, we can find the minimal rank *r* for each stack that satisfies the given threshold *β*, i.e., argmin_*r*_{*δ*_*r*_ < *β*}. Combining *δ*_*r*_ and *r*, the surrogate estimate for the amount of motion is given by *μ*_*r*_ = *δ*_*r*_∙*r*.

**Figure 1:**
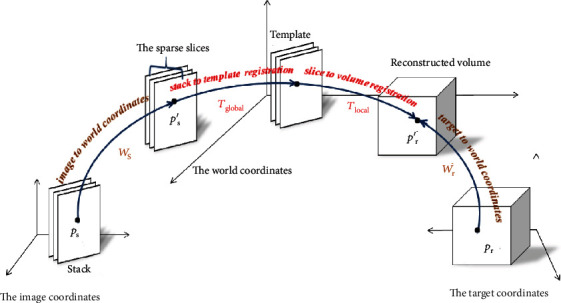
The illustration of the whole transformation process from pixels *p*_s_ to voxel *p*_r_.

Based on the low-rank decomposition method, we can choose one stack with minimal motion as the target template and first perform the 3D rigid volumetric registration between the target template and the other stacks (stack to template registration). During the first registration, we can get the corresponding rigid global transformation matrix *T*_global_. Then, second, the 3D rigid volumetric registration between the reconstructed volume and all slices (slice to reconstructed volume registration) can produce local transformation matrix *T*_local_. The prerequisite for two registrations is that all stacks and reconstructed volume should be mapped to the world coordinates. Thus, we need to define two transformations to map each pixel in the 2D slice and each voxel in the reconstructed volume to a continuous location in the world coordinates. The first one is world transformation *W*_s_ = [*θ*_1_^*w*^, ⋯, *θ*_*k*_^*w*^] that transforms the discrete coordinates of a pixel *p*_s_ = [*i*, *j*, 0, 1]^*T*^ ∈ *I*_*j*_^s^ in the acquired slice to the continuous local world coordinates. The second one is world transformation *W*_*r*_ = [*θ*_1_^*w*′^, ⋯, *θ*_*k*_^*w*′^] that transforms the discrete coordinates of a voxel *p*_r_ = [*x*, *y*, *z*, 1]^*T*^ ∈ *X* in the reconstructed volume to the continuous local world coordinates. Meanwhile, the mapping and registrations can be combined and formulated as Equation (4). Thus, Figure 1 illustrates the whole transformation process from the pixels in the sparse slice to voxels in the 3D reconstructed volume.

(4) $$\begin{array}{c} p_{\text{r}}=\left[W_{\text{r}}^{-1}\cdot T_{\text{total}}\cdot W_{\text{s}}\right]\cdot p_{\text{s}}=\left[W_{\text{r}}^{-1}\cdot \left(T_{\text{global}}\cdot T_{\text{local}}\right)\cdot W_{\text{s}}\right]\cdot p_{\text{s}}. \end{array}$$

### 2.2. PSF-Based Volume Update

To model the actual appearance of sampling data points in physical space, the point spread functions (PSFs) are used to make the exact estimation for every voxel value in the reconstructed target volume. For the MRI ssFSE sequence in this paper, the exact shape of the PSF has been measured using a phantom and rotating imaging encoding gradient in [14]. The resulting shapes of the PSF in in-plane and in through-slice are given by a sinc function and the slice profile, respectively. Since the ideal rectangle profile has the very dense and inefficient spatial sampling, Kuklisova-Murgasova et al. [28] have proposed to use the 3D Gaussian function with the full width at half maximum (FWHM) equal to the slice thickness as an approximation for the sinc function. The PSF function based on 3D Gaussian profile is used to approximately model the SSFSE sequence and is expressed as follows:

(5) $$\begin{array}{c} {\text{PSF}}_{\text{G}}=\exp\left(\frac{-{dx}^{2}}{2\sigma_{x}^{2}}+\frac{-{dy}^{2}}{2\sigma_{y}^{2}}+\frac{-{dz}^{2}}{2\sigma_{z}^{2}}\right), \end{array}$$

where *dx*, *dy*, and *dz* are the offsets from the center of a reconstructed voxel, *σ*_*x*_ and *σ*_*y*_ are the full width at half maximum (FWHM) in the in-plane *x-* and *y-*directions, and the *σ*_*z*_ equals to the slice thickness in the through-plane direction. For each pixel in the sampled slice, the PSF_G_ is applied to obtain the corresponding PSF coefficient matrix. Since every sampling pixel (i.e., *p*_s_) does not perfectly align itself with the reconstructed voxel (i.e., *p*_r_), one *p*_s_ contributes to more than one *p*_r_. To model this, every voxel is sampled around its local surrounding neighbor in the reconstructed volume to make sure that it has at least one corresponding pixel in the acquired slices. Then, the PSF coefficients are used to weigh the pixel's contribution during the*n*th iteration.

(6) $$\begin{array}{c} p_{\text{r}}=\left\lfloor \left(W_{\text{r}}^{-1}\cdot T_{\text{total}}\cdot W\right)\cdot p_{\text{s}}\right\rfloor ,\tilde{p_{\text{s}}}={\left(W_{\text{r}}^{-1}\cdot T_{\text{total}}\cdot W\right)}^{-1}p_{\text{r}},\\ \text{X}\left(p_{\text{r}}^{n+1}\right)=\text{PSF}\left(p_{\text{s}}-\tilde{p_{\text{s}}}\right)\cdot S_{j}^{\prime }\cdot \exp\left(-B_{j}\right)\cdot I_{j}^{i}\left(p_{\text{s}}\right)+X\left(p_{\text{r}}^{n}\right), \end{array}$$

where ⌊∙⌋ is the operation that finds the nearest voxel center in the space of the reconstructed volume. The reconstructed volume *X* is updated iteratively through the PSF-based data sampling model, and every voxel of *X* is filled at an arbitrarily chosen voxel size.

### 2.3. Robust Outlier Removal

Once the target volume is updated based on the Gaussian PSF, the simulated slices *I*^ss^ = [*I*_1_^ss^, ⋯, *I*_*k*_^ss^] ∈ **R**^*n*×*h*^ can be generated from the updated reconstructed volume. Then, the misaligned error *e*^∗^ between the corrected acquired sparse slices *I*′ and simulated slices *I*^ss^ can be computed as

(7) $$\begin{array}{c} E\left(e^{*}\right)=I^{\prime }\left(p_{\text{s}}\right)-I^{\text{ss}}\left(p_{\text{s}}\right). \end{array}$$

In [28], an EM model-based robust statistics approach was proposed to classify each slice pixel into two classes: inliers and outliers. Specially, the probability density function (PDF) for the inlier class is modeled as a zero-mean Gaussian distribution with variance *σ*^2^: *E* ~ *N*(0, *σ*^2^), and the PDF for the outlier class is modeled as a uniform distribution with constant density, which is a reciprocal of the range [*a*, *b*]: *E* ~ *U*(*a*, *b*). Then, the likelihood of the observing error *e*^∗^ can be expressed as

(8) $$\begin{array}{c} P\left(e^{*}\;|\;\sigma ,c\right)=c\cdot N_{\sigma }\left(e^{*}\right)+\left(1-c\right)\cdot U, \end{array}$$

where *c* is a mixing proportion of inliers representing the correctly matched voxels. Then, the posterior probability of a voxel being an inlier can be computed using the expectation step as

(9) $$\begin{array}{c} p_{ij}=\frac{c\cdot N_{\sigma }\left(e_{ij}^{*}\right)}{c\cdot N_{\sigma }\left(e_{ij}^{*}\right)+\left(1-c\right)\cdot U}. \end{array}$$

The variables *σ* and *c* are updated by the following maximization step:

(10) $$\begin{array}{c} \sigma =\sqrt{\frac{\sum p_{ij}\cdot {\left(e_{ij}^{*}\right)}^{2}}{\sum p_{ij}}},\\ c=\frac{\sum p_{ij}}{\sum N_{j}}, \end{array}$$

where *N* is the number of the pixels in the slice. By constantly iterating, we can get the best parameters *σ* and *c*. The inlier probability can be used to weigh the PSF-based volume update. By the same way, each slice is classified into inlier and outlier as well using the EM algorithm. The probability of an inlier slice is defined as $p_{j}^{\text{slice}}=\sqrt{\sum_{i}p_{ij}^{2}/N_{j}}$. The slices inferred to be an outlier are excluded from the PSF-based volume update to remove artifacts of motion corruption and misregistration.

The purpose of the outlier removal is to make the framework more robust by rejecting the outlier slices. The outlier removal module is adopted directly from the cited previous work [16], where the accuracy of the motion recognition and outlier removal has been evaluated in detail by simulating the slice motion at a variety of amplitudes and comparing the known motion amplitude to the surrogate measure provided through rank approximation. They have shown that there was strong correlation between the amplitude of the known motion and the values of *μ*_r_ derived from the stack data matrices.

### 2.4. Steering Kernel Regression-Based Volume Update

**Figure 2:**
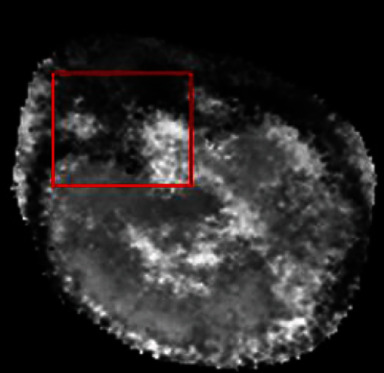
The reconstructed volume after PSF-based volume update.

For sparse reconstruction, it is experimentally found that the reconstructed volume still remains unallocated or inaccurate voxels after PSF-based volume update and the reconstructed result is noise as shown in Figure 2.

In [32], the kernel regression can make better nonparametric estimation for the empty pixels. In this paper, the steering kernel regression approach [29] is introduced to update the voxels for the previous sparse volume data. The model for the kernel regression is expressed as

(11) $$\begin{array}{c} Y_{i}=r\left(X_{i}\right)+\varepsilon_{i},\;i=1,\cdots ,M, \end{array}$$

where *r*(∙) is the function of kernel regression, *X*_*i*_ = (*x*_*i*_, *y*_*i*_, *z*_*i*_) is the 3D coordinate of the voxel, *ε*_*i*_ is a zero-mean Gaussian noise with variance *σ*_0_^2^ as *X* ~ *N*(0, *σ*_0_^2^), and *Y*_*i*_ is the voxel after PSF-based Gaussian volume update.

Assuming that the voxel *X*_*i*_ is close to the known voxel *X* in the reconstructed volume, we have the following approximation for *r*(*X*_*i*_) using the *N*-term-order Taylor series:

(12) $$\begin{array}{c} r\left(X_{i}\right)\approx r\left(X\right)+{\left\{\nabla r\left(X\right)\right\}}^{T}\left(X_{i}-X\right)+\frac{1}{2!}{\left(X_{i}-X\right)}^{T}\left\{Ηr\left(X\right)\right\}\left(X_{i}-X\right)+\cdots =\beta_{0}+\beta_{1}^{T}\left(X_{i}-X\right)+\beta_{2}^{T}\text{vech}\left\{\left(X_{i}-X\right){\left(X_{i}-X\right)}^{T}\right\}+\cdots , \end{array}$$

where ∇ and *Η* are, respectively, the gradient (3 × 1) and Hessian (3 × 3) operators; *β*_0_ = *r*(*X*), which is the voxel value of interest; and the vectors **β**_1_ and **β**_2_ are defined as

(13) $$\begin{array}{c} \beta_{1}={\left[G_{x},G_{y},G_{z}\right]}^{T}={\left[\frac{\partial r\left(X\right)}{\partial x},\frac{\partial r\left(X\right)}{\partial y},\frac{\partial r\left(X\right)}{\partial z}\right]}^{T}, \end{array}$$

(14) $$\begin{array}{c} \beta_{2}=\frac{1}{2}{\left[\frac{\partial^{2}r\left(X\right)}{\partial x^{2}},2\frac{\partial^{2}r\left(X\right)}{\partial x\partial y},2\frac{\partial^{2}r\left(X\right)}{\partial x\partial z},\frac{\partial^{2}r\left(X\right)}{\partial y^{2}},2\frac{\partial^{2}r\left(X\right)}{\partial y\partial z},\frac{\partial^{2}r\left(X\right)}{\partial z^{2}}\right]}^{T}. \end{array}$$

vech(∙) is the half-vectorization operator that transforms the upper triangular portion of a symmetric matrix into a column-stacked vector, i.e.,

(15) $$\begin{array}{c} \text{vech}\left(\left[\begin{array}{ccc} a & b & c\\ b & d & e\\ c & e & f \end{array}\right]\right)={\left[\begin{array}{cccccc} a & b & c & d & e & f \end{array}\right]}^{T}. \end{array}$$

Based on the least-squares formula, we can optimize Equation (12) as

(16) $$\begin{array}{c} \underset{{\left\{\beta_{n}\right\}}_{n=0}^{N}}{\min}\overset{L}{\underset{i=1}{\sum }}{\left[Y_{i}-\beta_{0}-\beta_{1}\left(X_{i}-X\right)-\beta_{2}{\left(X_{i}-X\right)}^{2}-\cdots \right]}^{2}\cdot \frac{1}{h}K\left(\frac{X_{i}-X}{h}\right), \end{array}$$

where *L* is the number of known voxels within the neighborhood window, *K*(∙) is the distance-weighted kernel function which penalizes distance away from the local position, and *h* is the smoothing parameter that controls the strength of the penalty. The kernel function is chosen as the exponential function, Gaussian function, or other functions which satisfy the following conditions:

(17) $$\begin{array}{c} \int tK\left(t\right)dt=0,\\ \int t^{2}K\left(t\right)dt=c. \end{array}$$

For the computation simplicity, the Gaussian-based kernel function is chosen in the steering kernel regression [33]. The steering kernel adapts locally to image structures (e.g., edges, flat, and texture areas), which are captured by the kernel footprint. For example, the kernel footprint is large in the flat areas, elongated in edge areas, and compact in texture areas. The 3D steering kernel function takes from

(18) $$\begin{array}{c} K_{\text{s}}\left({\text{X}}_{i}-X\right)=\frac{\sqrt{\det\left(C_{i}\right)}}{2\pi h^{2}}\exp\left\{-\frac{1}{2h^{2}}{\left\|C_{i}^{1/2}\left(X_{i}-X\right)\right\|}_{2}^{2}\right\}, \end{array}$$

where ‖∙‖_2_^2^ is the *L*_2_ norm and **C**_*i*_ is the symmetric covariance matrix. Since the local image structure is highly related to the gradient covariance, we can make the data-dependent covariance matrix estimation utilizing the local edge gradients:

(19) $$\begin{array}{c} {\hat{C}}_{i}\approx \left[\begin{array}{ccc} \underset{X_{i}\epsilon w}{\sum }G_{x}\left(X_{i}\right)G_{x}\left(X_{i}\right) & \underset{X_{i}\epsilon w}{\sum }G_{x}\left(X_{i}\right)G_{y}\left(X_{i}\right) & \underset{X_{i}\epsilon w}{\sum }G_{x}\left(X_{i}\right)G_{z}\left(X_{i}\right)\\ \underset{X_{i}\epsilon w}{\sum }G_{x}\left(X_{i}\right)G_{y}\left(X_{i}\right) & \underset{X_{i}\epsilon w}{\sum }G_{y}\left(X_{i}\right)G_{y}\left(X_{i}\right) & \underset{X_{i}\epsilon w}{\sum }G_{y}\left(X_{i}\right)G_{z}\left(X_{i}\right)\\ \underset{X_{i}\epsilon w}{\sum }G_{x}\left(X_{i}\right)G_{z}\left(X_{i}\right) & \underset{X_{i}\epsilon w}{\sum }G_{y}\left(X_{i}\right)G_{z}\left(X_{i}\right) & \underset{X_{i}\epsilon w}{\sum }G_{z}\left(X_{i}\right)G_{z}\left(X_{i}\right) \end{array}\right], \end{array}$$

where *w* is a local analysis window and *G*_*x*_(∙), *G*_*y*_(∙), and *G*_*z*_(∙) are the gradients along the *x*-, *y*-, and *z*-directions.

Equation (16) can be expressed in the matrix form as

(20) $$\begin{array}{c} \hat{B}=\underset{b}{\min}{\left\|Y-X_{F}B\right\|}_{W}^{2}=\underset{b}{\min}{\left(Y-X_{F}B\right)}^{T}W\left(Y-X_{F}B\right), \end{array}$$

where **Y** = [*Y*_1_, *Y*_2_, ⋯,*Y*_*L*_]^*T*^ is the vector set of all known voxels, **B** = [*β*_0_, **β**_1_^*T*^, ⋯,**β**_*N*_^*T*^]^*T*^ is the vector set of all estimated parameters, **W** = diag[*K*_s_(*X*_0_ − *X*), *K*_s_(*X*_1_ − *X*), ⋯, *K*_s_(*X*_*L*_ − *X*)] is the diagonal matrix whose elements on the diagonal are the value of *K*_s_(∙), and the other elements are zero. According to the least-squares method, we have the following solution:

(21) $$\begin{array}{c} \hat{B}={\left(X_{\text{F}}^{T}{WX}_{\text{F}}\right)}^{-1}X_{\text{F}}^{T}WY, \end{array}$$

where **B** = [*β*_0_, **β**_1_^*T*^, ⋯,**β**_*N*_^*T*^]^*T*^, $\hat{r}\left(X_{i}\right)={\hat{\beta }}_{0}=e_{1}^{T}{\left(X_{\text{F}}^{T}WX_{\text{F}}\right)}^{-1}X_{\text{F}}^{T}WY$ is the voxel value estimated by the steering kernel regression, ${\hat{\beta }}_{1}={\left[G_{x},G_{y},G_{z}\right]}^{T}$is applied for computing the symmetric covariance matrix ${\hat{C}}_{i+1}$ iteratively, and **X**_F_ is a coordinate matrix expressed as follows:

(22) $$\begin{array}{c} X_{\text{F}}=\left[\begin{array}{cccc} 1 & {\left(X_{0}-X\right)}^{T} & {\text{vech}}^{T}\left\{\left(X_{0}-X\right){\left(X_{0}-X\right)}^{T}\right\} & \cdots \\ 1 & {\left(X_{1}-X\right)}^{T} & {\text{vech}}^{T}\left\{\left(X_{1}-X\right){\left(X_{1}-X\right)}^{T}\right\} & \cdots \\ . & . & . & \cdots \\ . & . & . & \cdots \\ 1 & {\left(X_{L}-X\right)}^{T} & {\text{vech}}^{T}\left\{\left(X_{L}-X\right){\left(X_{L}-X\right)}^{T}\right\} & \cdots \end{array}\right]. \end{array}$$

Once the reconstructed volume is updated based on the steering kernel regression, we update the simulated slices *I*^ss^ and the misaligned error *E*(*e*^∗^) according to Equation (7). To remove artifacts caused by motion corruption and misregistration and enhance image edges, we further update the reconstructed volume using the following equation:

(23) $$\begin{array}{c} X\left(p_{\text{r}}^{n+1}\right)=\text{PSF}\left(p_{\text{s}}-\tilde{p_{\text{s}}}\right)\cdot p_{j}^{\text{slice}}\cdot p_{ij}\cdot E\left(e^{*}\right)+X\left(p_{\text{r}}^{n}\right). \end{array}$$

## 3. Implementation

**Figure 3:**
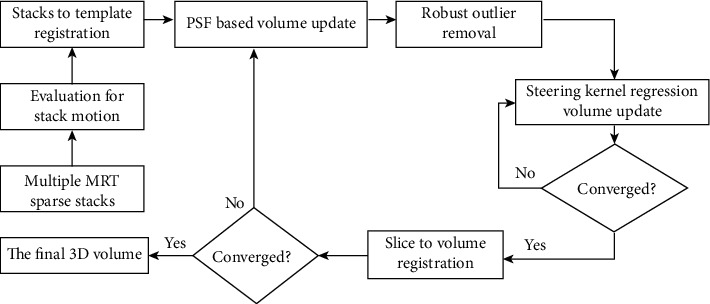
Flowchart of the proposed algorithm.

The experiment computer is equipped with Intel Core i5 2.6 GHz CPU, and the operating system is Windows 7 64 bit. We have implemented the proposed algorithm using the Microsoft Visual Studio 2012 and Image Registration Toolkit (IRTK) software package which includes many useful methods to do registration, transformation, and other image processing. In this section, we discuss the key implementation details. The diagram of the total algorithm is expressed in Figure 3.

**Figure 4:**
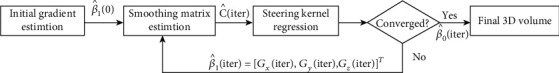
The iterative steering kernel regression.

The first step is to evaluate the stack motion according to the method of low-rank decomposition. We estimate the amount of the stack motion by the surrogate *μ*_*r*_ = *δ*_*r*_∙*r* and choose the stack with the minimum amount of stack motion as the template. The second step is to perform the global registration, which calculates the matrix of global transformation *T*_global_ from the other stacks to the template. The third step is the iterative registration-based volume reconstruction, which consists of the outer registration step and the inner reconstruction step. The outer loop step includes the PSF-based volume update, robust outlier removal, steering kernel regression-based volume update, and slice to volume registration. The PSF-based volume update step makes the initial estimation of the reconstructed volume based on Equation (6). Then, the simulated slices are created and used for the robust misaligned error calculation between the simulated slices and the acquired slices as described in Section 2.3. The robust statistic calculation achieves the classification of outlier slices and inlier slices. The outlier slices are excluded to remove artifacts of motion corruption and misregistration. The slice to volume registration is to calculate the local transformation *T*_local_ from slices to reconstructed volume. The whole transformation process is described by Equation (4). The volume update based on the adaptive steering kernel regression is aimed at reconstructing the accurate volume iteratively as shown in Figure 4. The initial gradients ${\hat{\beta }}_{1}\left(0\right)={\left[G_{x}\left(0\right),G_{y}\left(0\right),G_{z}\left(0\right)\right]}^{T}$ are estimated by the classical kernel regression. Then, the gradient information is used to calculate the covariance smoothing matrix $\hat{C}\left(\text{iter}\right)$ (i.e., Equation (19)). We use smoothing matrix to update the voxel value ${\hat{\beta }}_{0}\left(\text{iter}\right)$ and its corresponding gradients ${\hat{\beta }}_{1}\left(\text{iter}\right)$ according to Equation (21), respectively. To obtain a more reliable voxel estimation, the process is iterated three times in our experiment.

## 4. Experimental Results and Evaluation

### 4.1. Evaluation of Image Quality

**Figure 5:**
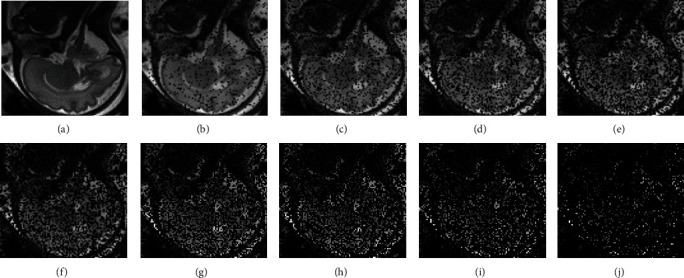
The original and spare slices: (a) the typical 30th original slice; (b–j) the corresponding simulated sparse slice by removing once every 10% proportion pixels ranging from 10% to 90%.

In the experimental evaluation, we used the datasets from the fetal MRI datasets [16], which were acquired by a Philips Achieva 3 T MR scanner. During the experiment, the volunteers were lying at a 20° tilt on the left side to avoid the pressure on the inferior vena cava. The volunteer's womb was scanned with single-shot fast spin echo (SSFSE) T2-weighted sequence. Three stacks of images from axial, coronal, and sagittal orientation are used to construct the final high-resolution volume. To obtain the sparse stacks, we randomly remove different proportions of pixels of the stack once every 10% proportion ranging from 10% to 90%. The different removal proportions control the removal number of pixels. The typical 30^th^ slice of the collected stack and its corresponding simulated spare slices are illustrated in Figure 5.

**Figure 6:**
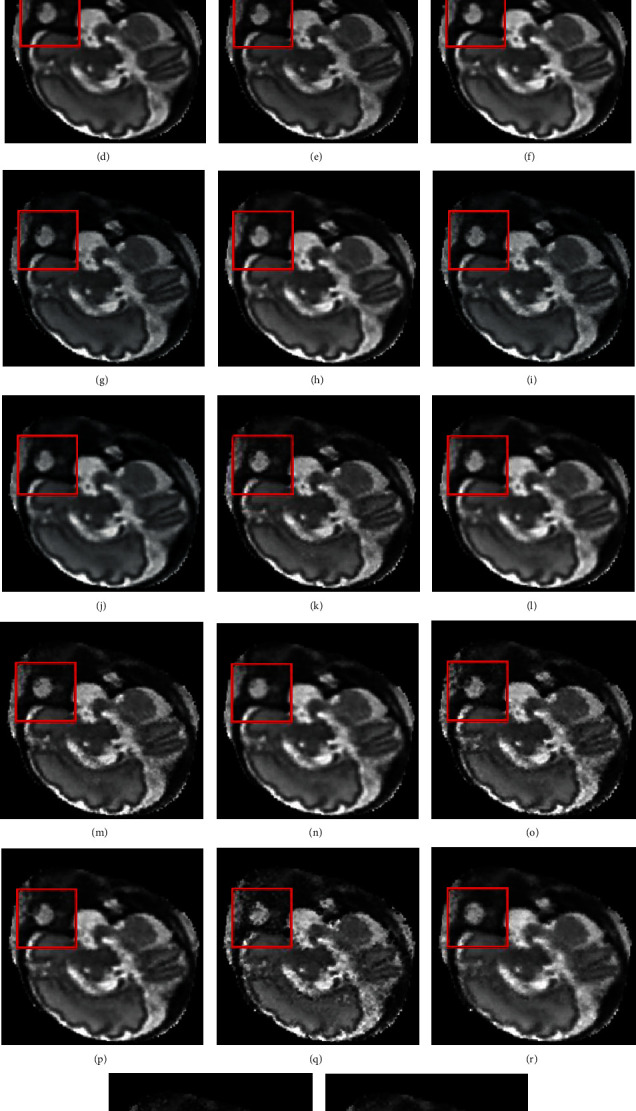
Reconstruction results of different data removal ratio by Kainz et al. method (2015) and our proposed method. (a), (c), (e), (g), (i), (k), (m), (o), (q), (s) are the reconstructed results by Kainz et al. method for the sparsely sampled dataset with once every 10% data removal ratio ranging from 0% to 90% respectively. (b), (d), (f), (h), (j), (l), (n), (p), (r), (t) are the reconstructed results by the proposed methodfor the sparsely sampled dataset with once every 10% data removal ratio ranging from 0% to 90%, respectively. The red rectangle points to the obvious difference, which appears as artifacts in the reconstructed image if no steering kernel regression volume updated is used.

For different data removal ratios, the sparse stacks are used to reconstruct the high-resolution 3D fetal brain MRI volume with the method of Kainz et al. [16] (SVR with superresolution) and our proposed method.  Figure 6 shows the reconstructed results by Kainz et al.'s method and the proposed method for the sparsely sampled dataset with once every 10% data removal ratio ranging from 0% to 90%, respectively. In Figure 6, we can observe that as the removal ratio increases, the reconstructed results by Kainz et al. method have much more noise for the sparse sampled dataset compared with our proposed method. On the other hand, the proposed method is capable of reconstructing high-resolution images without obvious artifacts even for the 90% data removal ratio.

For the sake of quantitative evaluation, the image quality assessment index of root mean square error (RMSE) [9] and mean structure similarity (MSSIM) [34] is introduced to quantitatively assess the algorithms under different removal ratios. The RMSE score can be computed by the following equation:

(24) $$\begin{array}{c} \text{RMSE}=\sqrt{\frac{1}{N}\overset{N}{\underset{i=1}{\sum }}{\left(\text{z}\left(i\right)-g\left(i\right)\right)}^{2}}, \end{array}$$

where *z*(∙) is the reconstructed result, *g*(∙) is the ground-truth volume, and *N* is the number of voxels. A good reconstruction method is capable of estimating the removal data very close to the original data. Given *z*(∙) and *g*(∙), a low RMSE value represents that the estimated result is satisfying while a high RMSE means that the interpolation accuracy is poor.

The structure similarity (SSIM) index explores the structural information for image quality assessment based on the main idea that the pixels have strong interdependency when they are spatially close. The SSIM metric is calculated based on the intensity, contrast, and structure and is computed as

(25) $$\begin{array}{c} \text{SSIM}\left(z,g\right)=\frac{\left(2\mu_{z}\mu_{g}+c_{1}\right)\left(2\sigma_{zg}+c_{2}\right)}{\left(\mu_{z}^{2}+\mu_{g}^{2}+c_{1}\right)\left(\sigma_{z}^{2}+\sigma_{g}^{2}+c_{2}\right)}, \end{array}$$

where *μ*_*z*_, *μ*_*g*_, *σ*_*z*_, *σ*_*g*_, and *σ*_*zg*_ denote the mean, variance, and covariance on square window, which moves pixel by pixel in images *z*(*i*) and *g*(*i*), respectively. The two variables *c*_1_ = *k*_1_*L* and *c*_2_ = *k*_2_*L* are used to stabilize the division with weak denominator. Here, *L* is the dynamic range of pixel value (e.g., 255 for 8-bit grayscale image), with *k*_1_ = 0.01 and *k*_1_ = 0.03 by default. Since the SSIM metric is calculated on various windows of a volume image, the mean SSIM (MSSIM) index is used in this experiment to assess the overall image quality:

(26) $$\begin{array}{c} \text{MSSIM}\left(z,g\right)=\frac{1}{M}\overset{M}{\underset{i=1}{\sum }}\text{S}\text{SIM}\left(z_{i},g_{i}\right), \end{array}$$

where *M* is the number of local windows in the image. MSSIM(*z*, *g*) ∈ [0, 1]; the higher MSSIM indicates better structural similarity between two images.

For the clinical datasets, it is impractical to obtain the ground-truth volume in advance. For the sake of fair comparison among different methods, the quantitative evaluation is performed based on an average reconstructed volume. We first use the original stacks without data removal to reconstruct a complete volume by Kainz et al.'s method (2015) and our method (e.g., Figures 6(a) and 6(b)), respectively. Both volumes are adopted to create an average volume as the ground truth.  Table 1 shows the quantitative results of the RMSE and MSSIM values with different data removal ratios for each method. As can be seen, the results of Kainz et al.'s method produce the highest RMSE scores and lowest scores for all sampling rates. Both the high RMSE value and low MSSIM value for Kainz et al.'s method indicate poor image quality because of the artifacts and noise. For all levels of sampling rate, the proposed method performs better than the Kainz et al.'s method. More importantly, both of the difference of the RMSE and MSSIM index between Kainz et al.'s method and our method increase while the data removal ratio increases, indicating that our method outperforms much more compared with the Kainz et al.'s method when the data removal ratio increases.

### 4.2. Evaluation of Computational Efficiency

Our approach is capable of reconstructing the accurate volume from the highly sparse sampling dataset, but it requires largely computational burden as well due to the iterative kernel regression estimation. To reduce the long processing time of the adaptive kernel regression, the proposed method is accelerated by the GPU-based parallel implementation based on the NVIDIA GeForce GTX 1080 and CUDA 8.0 libraries. In the experiment, we make the evaluation of the computational efficiency of the adaptive kernel regression method, including the computation of the gradient information, the covariance smoothing matrix, and the steering kernel regression. The computational efficiency of the other modules (i.e., motion estimation, stack-to-template registration, PSF-based volume update, robust outlier removal, and slice-to-volume registration) has been evaluated in detail in [16]. The comparisons are based on the single-threaded CPU, multithreaded CPU, and GPU for the dataset of 80% data removal ratio under the parameter setting as the kernel size *k*_c_ = 5 and the smoothing parameter *h*_c_ = 2.0 in the initial gradient estimation step based on the classical kernel regression, the steering kernel size *k*_s_ = 7 and the steering smoothing parameter *h*_s_ = 0.5 in the steering kernel regression step, and the window size *w* = 3, the regularization parameter *λ* = 2.0, and the structure sensitivity *α* = 0.4. The practical running time for the proposed method is shown in Table 2. For single-threaded CPU, the running time of the adaptive kernel regression method is 1865.769 s, which includes the computation of the gradient information in 416.96 s, the covariance smoothing matrix in 46.082 s, and the steering kernel regression in 1402.727 s. For the multithreaded CPU, we use 4 threads to run the adaptive kernel regression method and its computational time is 1045.293 s, indicating less improvement compared with the single-threaded CPUs. The running time of the GPU implementation is 20.718 s in total. From Table 2, it can be observed that the GPU-based processing time has significantly decreased by 98.89% and 98.02%, compared with the single-threaded CPU and the multithreaded CPU, respectively.

### 4.3. The Choice of the Adaptive Kernel Regression Parameters

There are seven parameters which can be adjusted to affect the reconstructed image quality for the proposed method. These parameters include the kernel size *k*_c_ and the smoothing parameter (i.e., the kernel bandwidth) *h*_c_ in the initial gradient estimation step based on the classical kernel regression, the steering kernel size *k*_s_ and the steering smoothing parameter *h*_s_ in the steering kernel regression step, and the window size *w*, the regularization parameter *λ*, and the structure sensitivity *α* (0 ≤ *α* ≤ 0.5) in the covariance matrix estimation step. In our method, *k*_c_ and *h*_c_ are related with the initial calculation of gradient information and have a negligible effect in the experiment. For the adaptive sparse reconstruction, covariance matrix estimation and steering kernel estimation are the two of the important steps and their parameters (i.e., *w*, *α*, *λ*, *k*_s_, and *h*_s_) play an important role in the volume reconstruction and deserve much more investigation.

**Figure 7:**
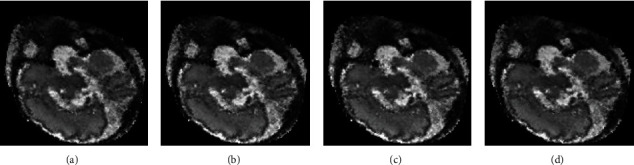
Reconstructed results of the MRI data with different window sizes *w*: (a) *w* = 3, (b) *w* = 5, (c) *w* = 7, and (d) *w* = 9.

With the help of GPU-based fast implementation, we firstly adjust the parameters (i.e., *w*, *α*, and *λ*) of the covariance matrix estimation one by one. The window size *w* decides how many neighbor points in the gradient matrix are taken for the estimation of the covariance matrix.  Table 3 shows the RMSE and MSSIM values and the running time for different window sizes. Both of the RMSE and MSSIM values differ slightly, indicating that the window size has a negligible influence on the reconstructed image quality, as shown in Figure 7. However, the running time increases with the increase of window size. It can be observed that the window size of *w* = 3 is chosen because of its faster implementation and lower RMSE value.

**Figure 8:**
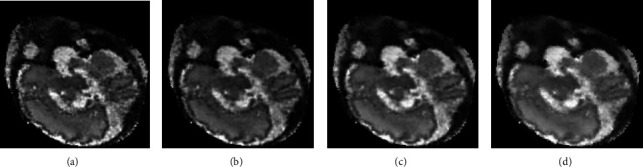
Reconstructed results of the MRI data with different structure sensitivities *α*: (a) *α* = 0.2, (b) *α* = 0.3, (c) *α* = 0.4, and (d) *α* = 0.5.

Table 4 shows the RMSE and MSSIM values influenced by the structure sensitivity parameter *α*, and the lowest RMSE value and highest MSSIM value are obtained for the structure sensitivity *α* = 0.4 indicating the best performance of the algorithm.  Figure 8 shows the corresponding reconstructed images for different *α* values. As can be seen, the result with large structure sensitivity (e.g., *α* = 0.5) results in oversmoothing image, while small structure sensitivity (e.g., *α* = 0.2) overemphasizes the image edges. The experiment shows that the structure sensitivity *α* has a significant influence on the reconstructed volume.

**Figure 9:**
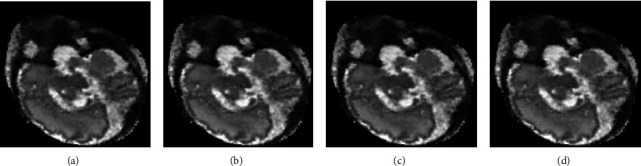
Reconstructed results of the MRI data with different regularization parameters *λ*: (a) *λ* = 0.5, (b) *λ* = 1.0, (c) *λ* = 1.5, and (d) *λ* = 2.0.

Under different regularization parameter settings, the RMSE and MSSIM measurements of the reconstructed results are calculated and shown in Table 5. The illustrative results are further shown in Figure 9. The regularization parameter *λ* is used to suppress the noise. However, the regularization parameter has negligible influence on the reconstructed image quality in the experiments.

**Figure 10:**
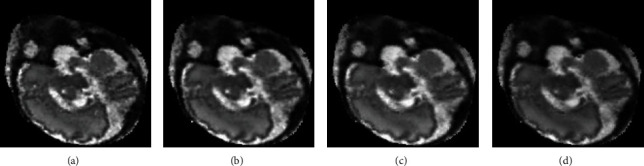
Reconstructed results of the MRI data with different kernel window sizes: (a) *k*_*s*_ = 3, (b) *k*_s_ = 5, (c) *k*_s_ = 7, and (d) *k*_s_ = 9.

The next group parameters (i.e., *k*_s_ and *h*_s_) come from the steering kernel regression for the adaptive voxel value estimation. The kernel window size *k*_s_ has a great impact on the processing time for the kernel regression-based algorithm under different data removal proportions. When the kernel window increases, the estimation of each voxel involves more nearby pixels and leads to more computation [32]. The smaller the kernel window size is, the faster our algorithm runs. On the other hand, if the size of the kernel window is too small, we could obtain the fault result, because there are not enough samples to make the current voxel estimation, especially for large data removal proportion. The larger the data removal proportion is, the sparser the sampled data will be. The RMSE and MSSIM index and processing time measurement of the reconstructed results under different kernel window sizes are shown in Table 6. With the increase of the kernel window size, the running time of steering kernel regression function is becoming longer. The corresponding images of different steering kernel sizes are shown in Figure 10. The proper kernel window size (i.e., 7 × 7 × 7) produces a trade-off between the processing time and the reconstruction accuracy under different removal proportions.

**Figure 11:**
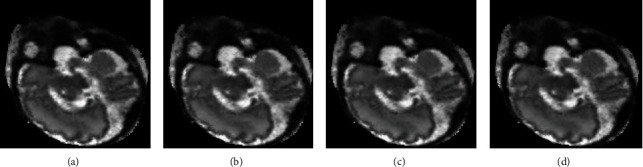
Reconstructed results of the MRI data with different steering smoothing parameters: (a) *h*_s_ = 0.5, (b) *h*_s_ = 1.0, (c) *h*_s_ = 1.5, and (d) *h*_s_ = 2.0.

Table 7 shows the RMSE and MSSIM values and running time with different steering smoothing parameters *h*_s_. As can be seen, the results with the steering smoothing parameter (i.e., *h*_s_ = 0.5) achieve the lowest RMSE value and highest MSSIM value among these settings. The reconstructed results produced by different steering smoothing parameters are shown in Figure 11. In [33], it has been given that the steering smoothing parameter indicates the “footprint” of the kernel function. The large footprint of the kernel function could reduce the noise but at the cost of oversmoothing details, while small footprints are desirable to preserve the edges. In the experiment, the footprint setting *h*_s_ = 0.5 is chosen for reaching a trade-off between the noise reduction and edge preservation. Finally, all parameters of the adaptive steering kernel regression algorithm are determined as follows: the window size *w* = 3, the regularization parameter *λ* = 2.0, the structure sensitivity *α* = 0.4, the steering kernel size *k*_s_ = 7, and the steering smoothing parameter *h*_s_ = 0.5. Under such parameter setting, the RMSE value decreases from 126.47 to 78.89, indicating the quality improvement by 37.62%.

## 5. Conclusion

In this paper, we proposed an adaptive reconstruction method to deal with the sparse sampling dataset for fetal brain MRI. Our method combines the latest SVR framework, including the stack motion evaluation, PSF-based volume update, robust outlier removal, slice-to-volume registration, and the proposed adaptive kernel regression-based volume update. Compared with the existing SVR framework, our method has advantages of sparse volume reconstruction and is capable of reconstructing superresolution image even for 80%~90% data removal. With the capability of sparse reconstruction, the data sampling time can be greatly shortened and thus, the motion artifacts can be reduced indirectly. To accelerate the voxel estimation, we use the CUDA to implement the steering kernel regression approach. For the proposed method, the running times of GPU-based implementation are speeded up to 90x than that of the CPU. The GPU-based parallel implementation of the proposed method is more practical to meet the requirements of fetal brain MRI. Meanwhile, we make the detailed investigation on the choice of parameters for the adaptive kernel regression-based volume reconstruction with the help of GPU-based fast implementation. To summarize, the structure sensitivity *α* and the steering kernel window size *k*_s_ are two of the important parameters on sparse kernel regression volume reconstruction. Meanwhile, the kernel window size has a strong relationship with the running time. Larger window size requires longer processing time. Overall, our approach is used to reconstruct superresolution image from the highly sparse sampled dataset of fetal brain MRI corrupted with motion artifacts. One of its potential applications includes other motion organ MRI reconstruction, such as the heart MRI with the heart beating motion artifacts.

## Figures and Tables

**Table 1:** The RMSE and SSIM value comparison of fetal brain reconstruction with different removal proportions, respectively.

Different removal proportions	RMSE		MSSIM	
	Kainz et al.'s method (2015)	Our method	Kainz et al.'s method (2015)	Our method
0%	19.096	19.096	1	1
10%	32.578	29.120	0.9752	0.9759
20%	38.043	33.947	0.9690	0.9691
30%	43.171	36.790	0.9591	0.9608
40%	49.194	41.027	0.9478	0.9458
50%	55.894	45.480	0.9202	0.9366
60%	67.053	53.305	0.9063	0.9271
70%	81.522	62.964	0.8667	0.8993
80%	111.917	78.886	0.7862	0.8449
90%	180.483	112.249	0.6338	0.7407

**Table 2:** The running time comparison of the adaptive kernel regression method for fetal brain reconstruction based on CPU and GPU, respectively.

Processor	Single-threaded CPU	Multithreaded CPU	GPU	Single-threaded CPU vs. GPU	Multithreaded CPU vs. GPU
Gradient information (s)	416.960	108.762	5.047	82.62	21.55
Covariance smoothing matrix (s)	46.082	48.902	0.580	79.45	84.31
Steering kernel regression (s)	1402.727	887.629	15.091	92.95	58.82
Total time (s)	1865.769	1045.293	20.718	90.06	50.45

**Table 3:** The RMSE and MSSIM values and running time comparison of fetal brain reconstruction with different window sizes ranging from 3 × 3 × 3 to 9 × 9 × 9, respectively.

Window size *w*	*w* = 3	*w* = 5	*w* = 7	*w* = 9
RMSE	125.061	125.869	129.298	126.929
MSSIM	0.6796	0.6663	0.6606	0.6763
TIME (s)	7.056 = (4.810 + 0.534 + 1.712)	10.268 = (4.822 + 3.714 + 1.732)	124.678 = (4.801 + 118.211^∗^ + 1.666)	222.353 = (4.798 + 215.888^∗^ + 1.667)

Note: TIME denotes the time caused only by running the adaptive kernel regression method. *T* = (*A* + *B* + *C*): *A* is the time to calculate the gradient information. *B* is the time to calculate the covariance smoothing matrix. *C* is the time to calculate steering kernel regression function. *T* is the sum of *A*, *B*, and *C*. ∗ indicates that CPU is chosen as the running processor for the covariance matrix calculation due to the limitation of GPU kernel memory for the large window size.

**Table 4:** The RMSE and MSSIM value comparison of fetal brain reconstruction with different structure sensitivities *α* ranging from 0.1 to 0.5, respectively.

Structure sensitivity	*α* = 0.1	*α* = 0.2	*α* = 0.3	*α* = 0.4	*α* = 0.5
RMSE	125.06	112.10	99.927	91.073	97.556
MSSIM	0.6823	0.7117	0.7584	0.7712	0.7523

**Table 5:** The RMSE and MSSIM value comparison of fetal brain reconstruction with the regularization parameter *λ* ranging from 0.1 to 2.0, respectively.

Regularization parameter *λ*	*λ* = 0.1	*λ* = 0.5	*λ* = 1.0	*λ* = 1.5	*λ* = 2.0
RMSE	91.073	91.447	91.276	91.566	91.071
MSSIM	0.7720	0.7710	0.7708	0.7684	0.7732

**Table 6:** The RMSE and MSSIM values and running time comparison of fetal brain reconstruction with different steering kernel sizes *k*_s_ ranging from 3 × 3 × 3 to 9 × 9 × 9, respectively.

Steering kernel size	*k*_s_ = 3	*k*_s_ = 5	*k*_s_ = 7	*k*_s_ = 9
RMSE	91.07	79.554	79.005	81.502
MSSIM	0.7791	0.7973	0.8054	0.7781
TIME (s)	7.907 = (5.407 + 0.581 + 1.919)	11.995 = (5.404 + 0.581 + 6.010)	21.148 = (5.402 + 0.581 + 15.165)	37.333 = (5.408 + 0.581 + 31.344)

Note: TIME denotes the time caused only by running the adaptive kernel regression method. *T* = (*A* + *B* + *C*): *A* is the time to calculate the gradient information. *B* is the time to calculate the covariance smoothing matrix. *C* is the time to calculate the steering kernel regression function. *T* is the sum of *A*, *B*, and *C*.

**Table 7:** The RMSE and MSSIM value comparison of fetal brain reconstruction with different steering smoothing parameters *h*_s_ ranged from 0.1 to 2.5, respectively.

Steering smoothing parameter	*h*_s_ = 0.1	*h*_s_ = 0.5	*h*_s_ = 1.0	*h*_s_ = 1.5	*h*_s_ = 2.0
RMSE	79.005	78.886	79.230	79.159	82.87
MSSIM	0.7927	0.8092	0.7933	0.7944	0.7946

## Data Availability

The test data was downloaded from the publicly available dataset on GitHub (https://github.com/bkainz/fetalReconstruction.git).
